# Control Circuits for Potentiostatic/Galvanostatic Polarization and Simultaneous Chemical Sensing by a Light-Addressable Potentiometric Sensor

**DOI:** 10.3390/s24175666

**Published:** 2024-08-30

**Authors:** Tatsuo Yoshinobu, Rintaro Ikeda, Ko-ichiro Miyamoto

**Affiliations:** 1 Department of Biomedical Engineering, Tohoku University, Sendai 980-8579, Japan; 2 Department of Electronic Engineering, Tohoku University, Sendai 980-8579, Japankoichiro.miyamoto.d2@tohoku.ac.jp (K.-i.M.)

**Keywords:** light-addressable potentiometric sensor, LAPS, pH sensor, chemical sensor, faradaic current, potentiostat, galvanostat, polarization, electrochemical system, corrosion

## Abstract

A light-addressable potentiometric sensor (LAPS) is a semiconductor-based sensor platform for sensing and imaging of various chemical species. Being a potentiometric sensor, no faradaic current flows through its sensing surface, and no electrochemical reaction takes place in the course of LAPS measurement. In this study, a four-electrode system is proposed, in which a LAPS is combined with the conventional three-electrode electrochemical system. A LAPS is included as the fourth electrode for potentiometric sensing and imaging of the target analyte in the course of an electrochemical reaction taking place on the surface of the working electrode. The integrated system will be useful for analyzing dynamic processes, where both the electrochemical process on the electrode surface and the ion distribution in the solution need to be simultaneously investigated. Different grounding modes of control circuits that can simultaneously conduct potentiostatic/galvanostatic polarization and LAPS measurement are designed, and their functionalities are tested. The interference between polarization and LAPS measurement will also be discussed.

## 1. Introduction

**Figure 1:**
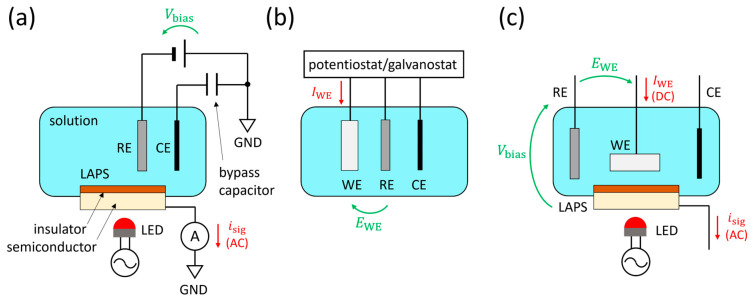
Schematics of electrode configurations of (**a**) a typical LAPS measurement system, (**b**) a conventional three-electrode system, and (**c**) a combined four-electrode system. An example of the real-space configuration of the four-electrode system is shown later in Section 4. LAPS, light-addressable potentiometric sensor; LED, light-emitting diode; RE, reference electrode; WE, working electrode; CE, counter electrode; GND, ground; AC, alternating current; DC, direct current.

A light-addressable potentiometric sensor (LAPS) [1,2,3,4,5,6,7] is a semiconductor-based chemical sensor in which the variation of the potential on the sensing surface in response to the analyte is detected in the form of an alternating current (AC) signal. Figure 1a shows a typical measurement setup of a LAPS, in which the sensor plate is virtually grounded via an ammeter that measures the AC signal *i*_sig_. The bias voltage *V*_bias_ applied between the reference electrode (RE) and the LAPS induces a depletion layer in the semiconductor, the width of which varies in response to the surface potential. Under this situation, the semiconductor layer is illuminated with a periodically modulated light beam, which generates the photocurrent signal *i*_sig_ to be correlated with the activity of the target analyte. The light-addressability is the most remarkable feature and the advantage of a LAPS, which makes it possible to conduct the measurement in a spatially resolved manner. Based on this feature, various chemical imaging sensor systems have been realized with a LAPS sensor plate scanned by a focused light beam [8,9,10,11,12,13,14].

A LAPS is a potentiometric sensor, in which no direct current flows through the insulated sensing surface, and no electrochemical reaction occurs as a result of the measurement. A LAPS measurement can, therefore, be said to be non-destructive in the sense that the measurement does not change the sample solution to be measured. On the other hand, it can also be a limitation because an electrode for a faradaic current to flow through is needed for analysis of a system in which an electrochemical reaction is taking place. There can be two approaches to realizing an electrochemical system with light-addressability. One approach is to combine a LAPS measurement system with a conventional three-electrode system, as shown in Figure 1b, resulting in a four-electrode system, as shown in Figure 1c. We previously employed such a configuration for pH imaging in the vicinity of a corroding metal surface under potentiostatic polarization [15,16,17]. The faradaic current flowing out from the working electrode (WE) will be collected by the counter electrode (CE), which also collects the AC signal from the LAPS. The other approach is to make the WE itself light-addressable. The light-addressable electrode (LAE) [18,19,20,21], the light-addressable amperometric sensor (LAAS) [22,23,24,25,26,27], the light-activated electrochemistry (also LAE) [28,29,30,31], and the photoelectrochemical imaging system (PEIS) [32,33,34] are examples of the latter approach, in which a faradaic current flows at the illuminated location on the electrode surface, which is not insulated.

This paper focuses on the circuit design of the four-electrode system mentioned above, which allows both potentiostatic and galvanostatic polarization of the WE and a simultaneous LAPS measurement in different grounding modes. Integration of LAPS in an electrochemical system allows a spatiotemporal interrogation of the ion distribution in the solution, in which an electrochemical reaction is taking place on the WE surface. The integrated system will be useful for analyzing dynamic processes, where both the electrochemical process on the electrode surface and the ion distribution in the solution need to be simultaneously investigated.

## 2. Circuit Design

### 2.1. Requirements

The basic requirements for the four-electrode system are as follows. For LAPS measurement, a bias voltage $V_{\mathrm{bias}}$ is applied between the RE and the LAPS so that the potential of the semiconductor substrate of the LAPS will be −*V*_bias_ vs. RE. The bias voltage *V*_bias_ is either constant or scanned in a typical range of −2 V to +2 V. As the surface of a LAPS is insulated, no direct current flows and only an AC photocurrent *i*_sig_ flows, which is typically of the order of μA. The frequency of *i*_sig_ is the same as the modulation frequency of the light beam, which is typically in the range of 100 Hz to 100 kHz. In potentiostatic polarization, the potential of the WE is controlled at a specified value *E*_WE_ vs. RE, and the resulting current flowing out of the WE, *I*_WE_, is monitored. In galvanostatic polarization, the current flowing out of the WE is controlled at a specified value *I*_WE_, and the resulting *E*_WE_ is monitored. In both cases, the target values are either constant or only slowly changing. The value of *E*_WE_ is typically in the range of −10 V to +10 V, whereas the magnitude of *I*_WE_ may very much differ depending on the system under polarization.

### 2.2. Grounding Modes

Two grounding modes of circuits are designed to meet different requirements of the electrochemical system and the LAPS measurement. In the first type of control circuit, the LAPS is virtually grounded via a transimpedance amplifier (TIA), which measures *i*_sig_. In most cases, this grounding mode is preferable because it is advantageous to stabilize the potential of the LAPS for the precise measurement of *i*_sig_, which is small. In the second type of control circuit, the WE is virtually grounded. This control circuit can be used when it is necessary, for some reason, to keep the WE at the ground potential (GND). The functions of the LAPS-grounded and the WE-grounded control circuits are identical except for their grounding modes.

### 2.3. Control Schemes

**Figure 2:**
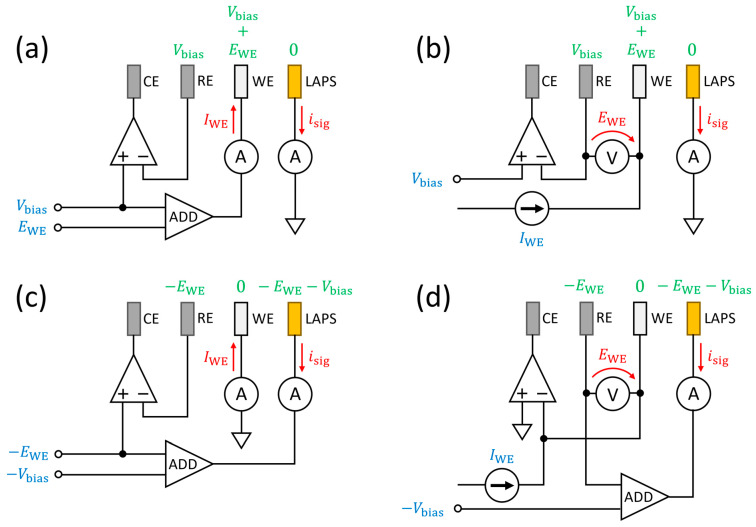
Simplified diagrams showing the control schemes of the four-electrode systems with different grounding modes for potentiostatic/galvanostatic polarization and simultaneous LAPS measurement. (**a**) A potentiostat with a virtually grounded LAPS. (**b**) A galvanostat with a virtually grounded LAPS. (**c**) A potentiostat with a virtually grounded WE. (**d**) A galvanostat with a virtually grounded WE. The values in blue and red show the input and output parameters, respectively, and the values in green show the potentials of the electrodes vs. GND. A unit labeled “ADD” is a voltage adder circuit.

Considering the combinations of the two grounding modes (LAPS-grounded and WE-grounded) and the two control modes (potentiostatic and galvanostatic), four control schemes were designed, as summarized in Figure 2. In these simplified diagrams, the values in blue and red show the input and output parameters, respectively. In all cases, *V*_bias_ is given as an input, and *i*_sig_ is read out as an output. *E*_WE_ and *I*_WE_ are input and output parameters, respectively, in a potentiostatic mode (PS mode) and vice versa in a galvanostatic mode (GS mode). The values in green show the potentials of the electrodes vs. GND.

The operation of each control circuit is briefly described below. Figure 2a,b shows simplified diagrams of the LAPS-grounded potentiostat and galvanostat, respectively. In these circuits, the LAPS is virtually grounded via an ammeter, and the potential of the RE is controlled at *V*_bias_ vs. GND by an operational amplifier. In Figure 2a, the potential of the WE is set at *V*_bias_ + *E*_WE_ vs. GND, or equivalently, *E*_WE_ vs. RE, and *I*_WE_ is measured. In Figure 2b, the WE is connected to a current source *I*_WE,_ and its potential *E*_WE_ vs. RE is monitored.

Figure 2c,d shows simplified diagrams of WE-grounded potentiostat and galvanostat, respectively. In Figure 2c, the WE is virtually grounded via an ammeter, which measures *I*_WE_, and the potential of the RE is controlled at −*E*_WE_ vs. GND by an operational amplifier so that the potential of the WE will be *E*_WE_ vs. RE. The potential of the LAPS is set at −*E*_WE_ − *V*_bias_ vs. GND so that the potential of the RE will be *V*_bias_ vs. LAPS. In Figure 2d, the WE is virtually grounded by an operational amplifier. The WE is also connected to a current source *I*_WE_, and *E*_WE_ is measured. The potential of the LAPS is set at −*E*_WE_ − *V*_bias_ so that the potential of the RE will be $V_{\mathrm{bias}}$ vs. LAPS.

## 3. Implementation

### 3.1. Potentiostat/Galvanostat with a Virtually Grounded LAPS

**Figure 3:**
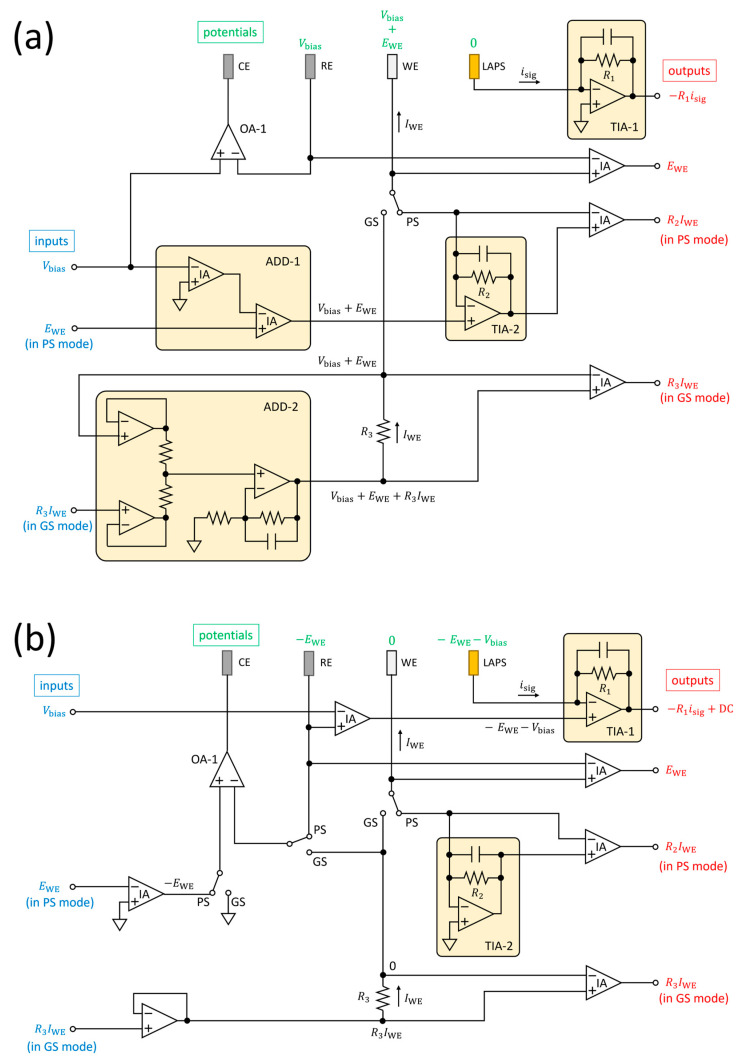
Diagrams of (**a**) LAPS-grounded and (**b**) WE-grounded control circuits for a four-electrode system, each of which can be switched between the PS mode and the GS mode. The power supply and the power supply bypass capacitors are omitted in the diagrams. The values in blue and red show the input and output parameters, respectively, and the values in green show the potentials of the electrodes vs. GND. All the instrumentation amplifiers are used with a unity gain, but those for monitor outputs can also be used with selective gains by connecting external resistances. OA, operational amplifier; IA, instrumentation amplifier; TIA, transimpedance amplifier; ADD, voltage adder circuit; PS, potentiostat; GS, galvanostat; GND, ground.

Figure 3a shows an example of a dual-functional circuit with a virtually grounded LAPS, which can be switched between the PS mode and the GS mode. The LAPS is virtually grounded by the TIA-1, which measures *i*_sig_ with a transimpedance gain determined by $R_{1}$. The TIA-1 can also be replaced with an external current preamplifier. In both the PS mode and the GS mode, the potential of the RE is controlled at *V*_bias_ by the operational amplifier OA-1. In the PS mode, the potential of the WE is set at *V*_bias_ + *E*_WE_ by the TIA-2, which measures *I*_WE_ with a transimpedance gain determined by *R*_2_. In the GS mode, the loop composed of the ADD-2 and *R*_3_ maintains the voltage difference across *R*_3_ at the specified value *R*_3_*I*_WE_ so that a current *I*_WE_ flows out of the WE.

The circuit was implemented on a printed circuit board (PCB) and operated with a power supply of ±12 V. The operational amplifiers used in TIAs were OP42GPZ (Analog Devices, Wilmington, MA, USA), and other operational amplifiers were OP07CPZ (Analog Devices, Wilmington, MA, USA). The instrumentation amplifiers LT1167CN8 (Linear Technology/Analog Devices, Wilmington, MA, USA) were used with a unity gain.

### 3.2. Potentiostat/Galvanostat with a Virtually Grounded WE

Figure 3b shows an example of a dual-functional circuit with a virtually grounded WE. In the PS mode, the WE is virtually grounded by the TIA-2, which measures *I*_WE_. The OA-1 controls the potential of the RE at −*E*_WE_ vs. GND so that the potential of the WE will be *E*_WE_ vs. RE. In the GS mode, the WE is virtually grounded by the OA-1. The current flowing out of the WE, *I*_WE_ is determined by the potential difference across *R*_3_. In both the PS mode and the GS mode, the TIA-1 controls the potential of the LAPS at −*E*_WE_ − *V*_bias_ so that the potential of the RE will be *V*_bias_ vs. LAPS. The TIA-1 measures *i*_sig_ with a transimpedance gain determined by *R*_1_. It should be noted that the output of the TIA-1 has a DC offset −*E*_WE_ − *V*_bias_, which is removed during the software lock-in detection of the amplitude of *i*_sig_.

The circuit was implemented on a PCB with the same chips as those used in the LAPS-grounded control circuit in Figure 3a. For unification of LAPS-grounded and WE-grounded control circuits, see Appendix A.

## 4. Measurement Setup

**Figure 4:**
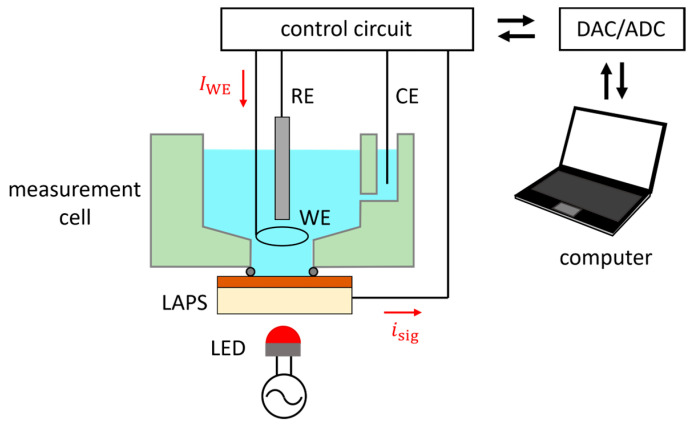
Schematic of the measurement cell for demonstration of potentiostatic/galvanostatic polarization and simultaneous LAPS measurement. The measurement cell is made of plexiglass, which accommodates 3 mL of test solution in contact with the LAPS surface. The four electrodes (LAPS, RE, WE, and CE) are connected to the control circuit developed in this study. DAC, digital-to-analog converter; ADC, analog-to-digital converter.

The functionalities of the developed circuits were tested in a measurement cell shown schematically in Figure 4. The measurement cell is made of plexiglass, which accommodates 3 mL of test solution, an Ag/AgCl RE (RE-1B, BAS Inc., Tokyo, Japan), and two Pt wires as the WE and the CE. The bottom of the measurement cell has an opening with a diameter of 10 mm, to be sealed with the LAPS surface with an O-ring. The distance between the WE and the LAPS surface is 5 mm. The LAPS sensor plate has the same layer structure as those used in our previous studies [17]; it is made of n-type Si with a resistivity of 1–10 Ωcm, with a size of 17.5 mm × 17.5 mm and a thickness of 200 μm. A 50 nm thick thermal oxide and a 50 nm thick Si_3_N_4_ are deposited in this order on the front surface, and a gold electrode is evaporated on the rear surface. As a light source for exciting photocarriers, a visible light-emitting diode (LED) is advantageous for the ease of operation and the availability of ultrahigh brightness. In our setup, a red LED with a peak wavelength of 630 nm (L5-EKR2530-12500, Full Sun Optotech, Taichung City, Taiwan) shines on the rear surface of the LAPS to generate a photocurrent signal. The LED current is sinusoidally modulated at 2500 Hz with peak currents being 0 and 20 mA. The modulation signal is supplied by a digital function generator (DF1906, NF Corporation, Yokohama, Japan), which has a frequency accuracy of 25 ppm. The input/output parameters of the control circuit are supplied/read out by a 16-bit digital-to-analog/analog-to-digital converter (DAC/ADC, USB-6002, National Instruments/Emerson Electric, Austin, TX, USA), which has a timebase accuracy of 100 ppm. The LAPS signal is digitized at a sampling rate of 50 kHz, and the amplitude of its frequency component at 2500 Hz is numerically calculated from a sequence of 10,000 sampling points obtained in 200 ms. Considering the high accuracies of both the modulation frequency and the sampling frequency, the software lock-in detection internally generates sine and cosine functions of the reference frequency based on the sampling period instead of a reference signal from the digital function generator. The experiments were conducted using homemade software written with LabVIEW^®^ 2024 (National Instruments/Emerson Electric, Austin, TX, USA).

## 5. Results and Discussion

### 5.1. Current-Voltage Curves of LAPS

**Figure 5:**
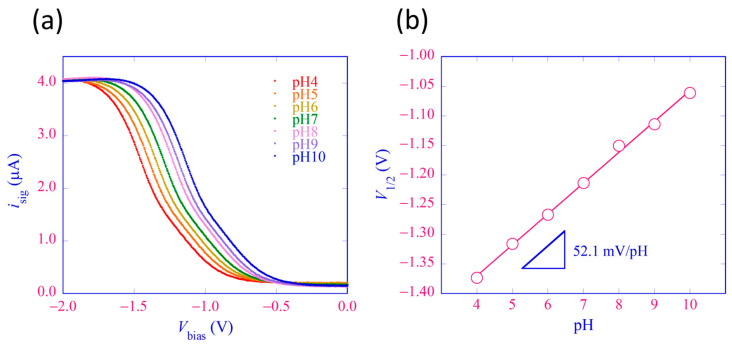
Characterization of the LAPS sensor plate using the LAPS-grounded control circuit in Figure 3a operated in the PS mode. The value of *E*_WE_ was set to 0 V vs. RE during the measurement. (**a**) The current-voltage (*i*_sig_–*V*_bias_) characteristics measured for a series of pH buffer solutions with different pH values from 4 to 10. (**b**) A calibration plot of the LAPS sensor plate. The value of the bias voltage *V*_1/2_, at which the LAPS signal *i*_sig_ equal to half the maximum, is plotted as a function of pH.

The current-voltage curves of the LAPS sensor plate were obtained using the LAPS-grounded control circuit operated in the PS mode. The bias voltage *V*_bias_ applied to the RE with respect to the LAPS was varied in the range of −2 to 0 V with a step of 5 mV, which corresponded to the transition of the state of the insulator–semiconductor interface from inversion to depletion to accumulation. After each stepwise change of *V*_bias_, sampling of *i*_sig_ was started after a delay of 50 ms to ensure completion of charging/discharging the capacitance of the LAPS sensor plate under the updated bias. The value of *E*_WE_ vs. RE was fixed at 0 V so that there was no faradaic current throughout this measurement. Figure 5a shows the *i*_sig_–*V*_bias_ curves for a series of pH buffer solutions (Titrisol^®^, Merck, Darmstadt, Germany) with pH 4 to 10. A horizontal shift of the curves along the voltage axis was observed, indicating the pH dependence of the potential difference between the sensing surface and the solution. For each curve, the bias voltage *V*_1/2_, at which the current became half the maximum, was calculated and plotted versus pH, as shown in Figure 5b, which gave the slope sensitivity of this LAPS sensor plate 52.1 mV/pH.

### 5.2. Polarization and LAPS Measurement with LAPS-Grounded Control Circuit

**Figure 6:**
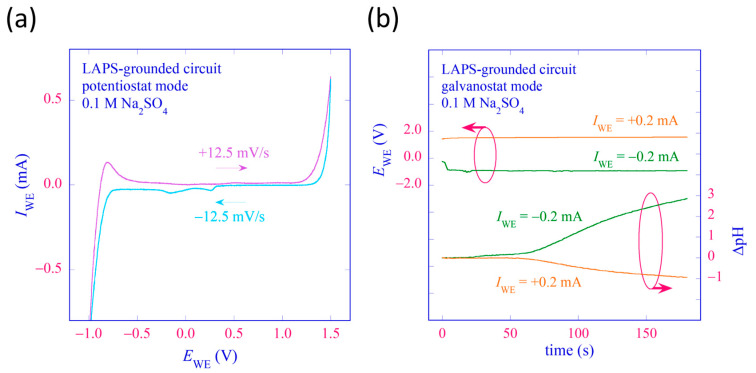
Results of polarization experiments conducted and recorded with a LAPS-grounded control circuit. The test solution was 0.1 M Na_2_SO_4_, and the WE was a Pt wire placed at a distance of 5 mm from the LAPS surface, as shown in Figure 4. (**a**) A polarization curve recorded in the PS mode. (**b**) Simultaneous recording of *E*_WE_ and ΔpH in the course of galvanostatic polarization with *I*_WE_ = ±0.2 mA.

An example of a polarization curve was obtained using the LAPS-grounded control circuit operated in the PS mode. The test solution was 3 mL of 0.1 M Na_2_SO_4_. The value of *E*_WE_ was varied between −1 V and +1.5 V at a rate of ±12.5 mV/s in both directions, and *I*_WE_ was recorded. Figure 6a shows the obtained *I*_WE_–*E*_WE_ curves, in which electrolysis of water is observed at higher and lower potentials.

The LAPS-grounded control circuit was then operated in the GS mode, and the WE was galvanostatically polarized both anodically and cathodically using the same test solution. The value of *I*_WE_ was controlled at ±0.2 mA, and the value of *E*_WE_ during galvanostatic polarization was recorded. At the same time, *i*_sig_ was recorded under a fixed value of *V*_bias_ = −1.3 V, which was in the middle of the transition region of the *i*_sig_–*V*_bias_ curve of the test solution. The variation of *i*_sig_ was converted into the potential shift using the slope of the *i*_sig_–*V*_bias_ curve, −7.03 μA/V at *V*_bias_ = −1.3 V, and the potential shift was further converted into the pH change using the slope sensitivity of the LAPS sensor plate, 52.1 mV/pH. For a detailed discussion of this conversion, see Appendix B. Figure 6b shows the result of this experiment. When the WE was an anode, the pH value decreased due to the anodic reaction: 2H_2_O→O_2_ + 4H^+^ + 4e^−^ after an initial delay of ca. 60 s. The initial delay was the time needed for protons generated at the WE to reach the LAPS surface by diffusion, which was verified by coloring the solution with methyl orange. Contrarily, when the WE was a cathode, the pH value increased due to the cathodic reaction: 2H_2_O + 2e^−^→H_2_ + 2OH^−^. These results demonstrated the functionalities of the LAPS-grounded control circuit, which can polarize the WE both potentiostatically and galvanostatically, and record the resulting pH change on the LAPS surface at the same time.

### 5.3. Polarization and LAPS Measurement with WE-Grounded Control Circuit

**Figure 7:**
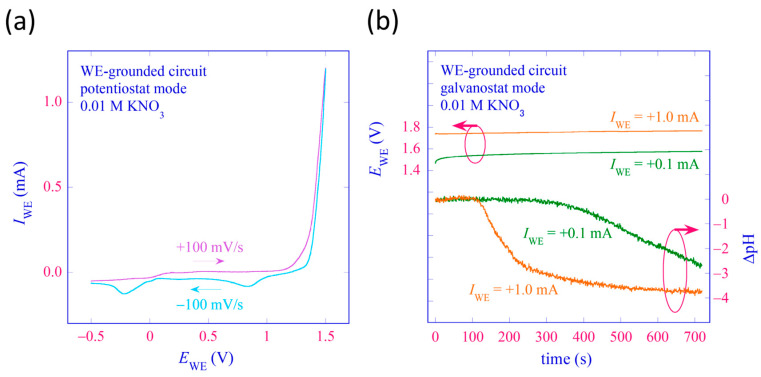
Results of polarization experiments conducted and recorded with a WE-grounded control circuit. The test solution was 0.01 M KNO_3_, and the WE was a Pt wire placed at a distance of 5 mm from the LAPS surface, as shown in Figure 4. (**a**) A polarization curve recorded in the PS mode. (**b**) Simultaneous recording of *E*_WE_ and ΔpH in the course of galvanostatic polarization with *I*_WE_ = +0.1 mA and +1.0 mA.

Similar experiments were also carried out with a WE-grounded control circuit to test its functionality. This time, the test solution was 3 mL of 0.01 M KNO_3_. Figure 7a shows an example of a polarization curve obtained by varying *E*_WE_ in the PS mode at a rate of ±100 mV/s in the range between −0.5 V and +1.5 V. Electrolysis of water is observed at higher potentials. Figure 7b shows the temporal changes of *E*_WE_ and ΔpH during anodic polarization of the WE in the GS mode with *I*_WE_ = +0.1 mA and +1.0 mA. The value of *V*_bias_ for LAPS measurement was fixed at −1.4 V. Lowering of pH due to the anodic reaction at different rates dependent on *I_WE_* was observed, demonstrating the functionality of the WE-grounded control circuit.

### 5.4. Interference between Polarization and LAPS Measurement

In this section, the possibility of interference between polarization and LAPS measurement is discussed. In the four-electrode system, both *I*_WE_ and *i*_sig_ are ideally collected entirely by the CE, which has a low input impedance. When the WE and the LAPS are placed in close proximity, however, there may be crosstalk between them.

As the LAPS surface is insulated, the DC component of the current flowing out of the WE, *I*_WE_ will not enter the LAPS and will be collected only by the CE. When *I*_WE_ contains an AC component, either due to fluctuation of the electrochemical reaction on the WE surface or due to electrical noise, it may enter the LAPS via capacitive coupling between the solution and the semiconductor layer of the LAPS. Its influence on the measurement of *i*_sig_ can, however, be mostly eliminated during the software lock-in detection of *i*_sig_ at the frequency of modulation. Therefore, the influence of polarization on the LAPS measurement can be ignored in most cases.

Conversely, the alternating current flowing out of the LAPS surface, −*i*_sig_ in our notation, can enter the WE when it is placed in close proximity to the LAPS surface. In the GS mode, the control circuit has a high impedance at the output terminal connected to the WE and, therefore, can reject this crosstalk. In the PS mode, however, the control circuit has a low impedance at the output terminal, and the current enters the circuit. Whether this crosstalk can be a problem or not depends on the relative magnitudes of *I*_WE_ and *i*_sig_. Additionally, a coil may be inserted in series with the WE to reject an alternating current.

Yet another form of interference may occur when *V*_bias_ is changed during measurement. The bias voltage applied between the RE and the LAPS, *V*_bias_, is either kept constant or varied depending on the purpose of LAPS measurement. When the temporal resolution has priority, *V*_bias_ is fixed, and the temporal change of *i*_sig_ is recorded and converted into ΔpH as was done in Figure 6b and Figure 7b. When the precision in determining the activity of the analyte is prioritized, *V*_bias_ is varied in a certain range to obtain the *i*_sig_–*V*_bias_ curve such as the one in Figure 5a, from which the voltage shift can be more precisely calculated. In the latter case, *V*_bias_ is changed stepwise during LAPS measurement. In the case of measurement in Figure 5a, for example, the step was 5 mV. When *V*_bias_ is abruptly changed, there will be a transient current charging/discharging the capacitance of the LAPS. In addition, it will disturb the feedback loop controlling *E*_WE_ in the PS mode or *I*_WE_ in the GS mode until the fluctuation is settled down.

**Figure 8:**
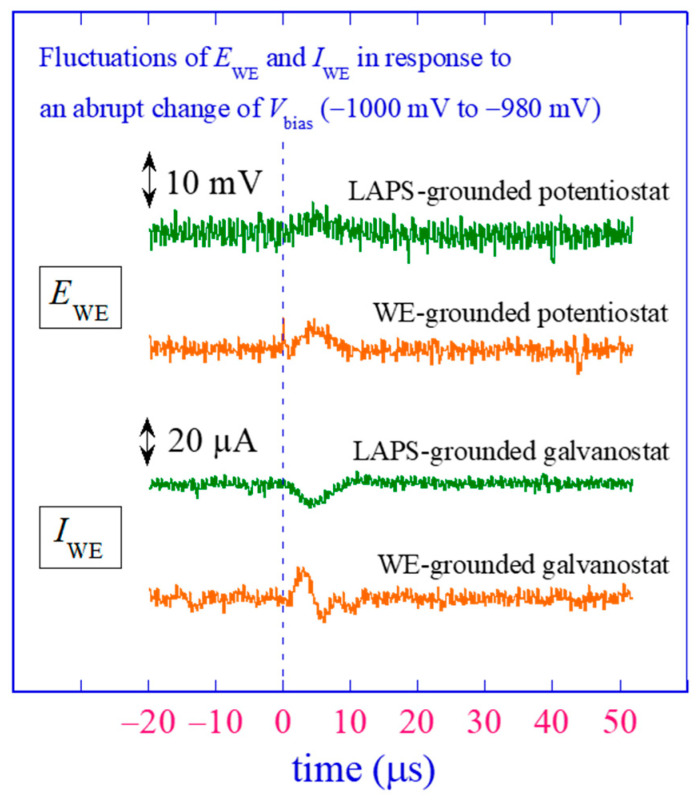
Assessment of the influence of LAPS measurement on polarization. The target values of *E*_WE_ (in the PS mode) and *I*_WE_ (in the GS mode) were set at 0, and the value of *V*_bias_ applied between the RE and the LAPS was abruptly changed from −1000 mV to −980 mV. Fluctuations of *E*_WE_ and *I*_WE_ in response to this stepwise change of *V*_bias_ were recorded at a sampling rate of 5 MS/s.

The influence of the change of *V*_bias_ on the potentiostatic/galvanostatic polarization was experimentally assessed using the developed circuits. The test solution was 3 mL of 0.1 M KNO_3_, and the target values of *E*_WE_ in the PS mode and *I*_WE_ in the GS mode were set at 0. Then, *V*_bias_ was abruptly changed from −1000 mV to −980 mV by a step of 20 mV, and the resulting fluctuations of *E*_WE_ and *I*_WE_ were recorded at a sampling rate of 5 MS/s using a digital storage oscilloscope (MSO22, Tektronics, Beaverton, OR, USA) as shown in Figure 8. The disturbance continued for ca. 10 μs until the control recovered. The fluctuations of *E*_WE_ in the PS mode and *I*_WE_ in the GS mode were ca. 10 mV and 20 μA, respectively. These values are small enough for corrosion experiments [15,16,17], but if a higher precision is required, *V*_bias_ must be changed by a smaller step. It should be noted that the duration and the magnitude of disturbance would depend on the details of the electrochemical system as well as the implementation of the control circuit.

## 6. Conclusions

In this study, a four-electrode system was proposed, in which a LAPS was included as the fourth electrode for potentiometric sensing of the target analyte in the course of an electrochemical reaction taking place on the surface of the WE. Control circuits with different grounding modes that can simultaneously conduct potentiostatic/galvanostatic polarization and LAPS measurement were designed and developed, and their functionalities were verified in a demonstration of the temporal recording of pH change due to anodic/cathodic reactions in electrolysis of water. The possibility of interference between polarization and LAPS measurement was discussed in detail.

The integration of a LAPS into a conventional three-electrode system will allow simultaneous recording of the electrochemical reaction on the WE surface and the spatiotemporal distribution of the target analyte in the vicinity of the WE surface. This measurement system is expected to be useful for the analysis of dynamics involving not only electrochemistry but also reaction and diffusion in the solution.

Finally, one of the interesting applications of the proposed four-electrode system would be the combination of amperometric and potentiometric sensors [35,36,37,38,39,40], using an amperometric sensor as the WE and a LAPS as the fourth electrode. For example, the enzymatic oxidation of glucose catalyzed by glucose oxidase produces gluconic acid and hydrogen peroxide. While the latter is amperometrically detected, the pH change due to the former can be recorded to correct for the influence of pH. For such applications, where *I*_WE_ can be even smaller than *i*_sig_, the design and implementation of the control circuit would require further sophistication and optimization.

## Figures and Tables

**Figure A1:**
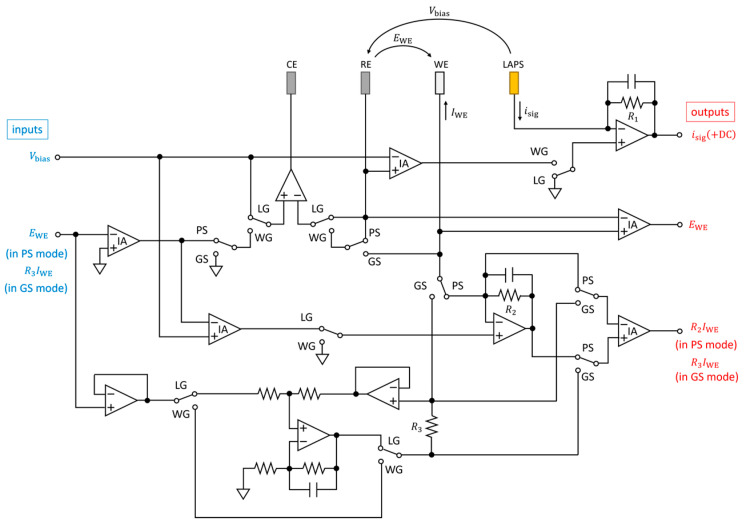
A unified control circuit that can be switched between the LAPS-grounded mode and the WE-grounded mode. In each grounding mode, the operation mode can be switched between the PS mode and the GS mode. LG, LAPS-grounded; WG, WE-grounded; PS, potentiostat; GS, galvanostat; IA, instrumentation amplifier.

## Data Availability

Data are contained within the article.

## Appendix A

Taking advantage of the similarities between the LAPS-grounded and WE-grounded control circuits in Figure 3, they can be unified, as shown in Figure A1.

## Appendix B

In the polarization experiments shown in Figure 6b and Figure 7b, the photocurrent signal *i*_sig_ was recorded under a constant value of *V*_bias_, and the temporal variation of *i*_sig_ was converted into ΔpH using the slope of the transition region of the *i*_sig_–*V*_bias_ curve such as the one shown in Figure 5a. Here, the linearity of the transition region was implicitly assumed, which, in fact, is not accurate when the variation of *i*_sig_ is not small. Particularly, as can be seen in Figure 5a, the slope becomes considerably smaller as the current decreases. The actual lowering of pH is, therefore, larger than those shown in Figure 6b and Figure 7b, which also explains the asymmetry of ΔpH curves for anodic and cathodic polarization in Figure 6b. For a more accurate conversion of *i*_sig_ into ΔpH, the shape of the *i*_sig_–*V*_bias_ curve must be taken into account.
